# Effects of 2 or 5 consecutive exercise days on adipocyte area and lipid parameters in Wistar rats

**DOI:** 10.1186/1476-511X-6-16

**Published:** 2007-07-02

**Authors:** Ricardo LF Guerra, Wagner L Prado, Nádia C Cheik, Fabiana P Viana, João Paulo Botero, Regina C Vendramini, Iracilda Z Carlos, Elizeu A Rossi, Ana R Dâmaso

**Affiliations:** 1 Federal University of São Paulo – Baixada Santista, Health Science Center, Santos, Brazil; 2 CCBS/Federal University of São Carlos, Department of Physiology Sciences, São Paulo, Brazil; 3 FCF/Paulista State University of Araraquara, Department of Food and Nutrition, São Paulo, Brazil

## Abstract

**Background:**

Exercise has been prescribed in the treatment and control of dyslipidemias and cholesterolemia, however, lipid responses to different training frequencies in hypercholesterolemic men have been inconsistent. We sought to verify if different frequencies of continuous moderate exercise (2 or 5 days/week, swimming) can, after 8 weeks, promote adaptations in adipocyte area and lipid parameters, as well as body weight and relative weight of tissues in normo and hypercholesterolemic adult male rats.

**Methods:**

Normal cholesterol chow diet or cholesterol-rich diet (1% cholesterol plus 0.25% cholic acid) were freely given during 8 weeks to the rats divided in 6 experimentals groups: sedentary normal cholesterol chow diet (C); sedentary cholesterol-rich diet (H); 5× per week continuous training normal cholesterol chow diet (TC5) and cholesterol-rich diet (TH5); 2× per week continuos traning normal cholesterol chow diet (TC2) and cholesterol-rich diet (TH2).

**Results:**

No changes were observed in lipid profile in normal cholesterol chow diet, but both 2 a 5 days/week exercise improved this profile in cholesterol-rich diet. Body weight gain was lower in exercised rats. Decrease in retroperitoneal and epididymal relative weights as well as reductions in adipocyte areas under all diets types were observed only in 5 days/week, while 2 days/week showed improvements mainly in cholesterol-rich diet rats.

**Conclusion:**

Our results confirm the importance of exercise protocols to control dyslipidemias and obesity in rats. The effects of 5 days/week exercise were more pronounced compared with those of 2 consecutive days/week training.

## Background

Although vital to the body as a structural component of membranes and for the biosynthesis of steroid hormones, cholesterol exceeding ideal limits can result in several health problems such as: atherosclerosis (which can cause angina, strokes, and heart attacks), dyslipidemias, obesity, etc, leading to death in some cases [1-3]. It is known that the body can obtain cholesterol through exogenous sources and that humans are capable of endogenous synthesis. Most dyslipidemias and hypercholesterolemia are regarded as consequences of hypoactivity and bad eating habits caused by a lifestyle characterized by fat-rich diets and high cholesterol levels [4]. On the other hand, increased exercise practice, mainly continuous and aerobic, is considered one of the fundamentals in preventing and treating these diseases [5,6]. Coronary risk is twice as large in sedentary people than it is in those who are active; furthermore, physical inactivity can contribute to arterial hypertension and low levels of high density lipoprotein cholesterol (HDL-c) and hypertriglyceridemia, factors associated with atherosclerotic diseases. In contrast, exercise can raise aerobic fitness levels and improve lipid oxidative capacity. It can prevent coronary heart diseases (CHD) by decreasing adipocyte volume, and plasmatic concentrations of insulin, triglycerides, and low density lipoprotein (LDL-c), while increasing HDL-c [7,8]. Furthermore, the few available studies reporting lipid and lipoprotein responses to exercise training in hypercholesterolemic men indicate that reductions in total cholesterol (TC) and triglycerides (TG), as well as elevations in HDL-C are also possible. These results are somewhat similar to those reported in normolipidemic subjects; however, lipid responses to exercise training and different training frequencies in hypercholesterolemic men have been inconsistent and, at present, there are too few studies from which to draw general conclusions [9].

Thus, despite several studies on dyslipidemias and obesity control, sedentary habits associated with consumption of high-cholesterol diets continue to cause negative effects on public health, mainly in adult males. To contribute towards reverting this situation, we have compared for the first time the effect of 5 or 2 days/week consecutive exercise days on adult male rats feed with normal cholesterol chow diet and cholesterol-rich diet to see whether these different frequencies of continuous moderate exercise could promote adaptations in the adipocyte area and lipid parameters.

## Results

Total food intake increased statistically in all cholesterol-rich diet groups compared to control groups (Table 1). There were no significant differences between sedentary and trained groups or between different frequencies of exercise in rats fed with the same diet.

**Table 1:** Body weight gain (%) and total food intake (g) in normal cholesterol chow diet and cholesterol-rich diet rats submitted to continuous moderate training (5 or 2 consecutive days/week) during 8 weeks

**Group/Tissue**	**Total Food Intake (g)**	**Body Weight Gain (%)**
**C**	1108 ± 55.05	64.06 ± 4.18
**TC5**	1127 ± 34.73	36.58 ± 2.88 *+
**TC2**	1034 ± 55.70	60.98 ± 3.01
**H**	1405 ± 33.64 °	94.86 ± 3.64 °
**TH5**	1347 ± 23.19 °	68.35 ± 3.53 *°
**TH2**	1252 ± 31.22 °	72.20 ± 4.38 *

* p ≤ 0.05 comparing trained × sedentary groups; +p ≤ 0.05 comparing 5 × 2 days/week exercised groups; ° p ≤ 0.05 comparing normal cholesterol chow diet × cholesterol-rich diet groups. Sedentary control (**C**) and cholesterol-rich diet group (**H**); 5 and 2× per week continuous training control (**TC5**) (**TC2**); 5 and 2× per week continuous training cholesterol-rich diet group (**TH5**) **(TH2).**

However, sedentary cholesterol-rich diet rats showed significant increase in body weight gain (Table 1, CxH°). Five days/week exercise in both control and the cholesterol-rich diet group effectively decreased statistically body weight gain, whereas 2 days/week exercise was only effective in body weight gain reduction in cholesterol-rich diet rats. Comparing exercise frequencies, significant body-weight gain modification was only observed in control rats trained 5 days/week.

A significant TC and plasma TG is noted higher in comparing the H (108.70 ± 5.22 °**; **177.83 ± 15.78 °) to the C group (62.17 ± 3.07; 122.67 ± 15.09) (Table 2). Both continuous 5 and 2 days/week exercise frequencies were able to promove lower TC and TG in rats submitted a cholesterol-rich diet (*). These results were not observed in control rats, for whom different frequencies of exercise presented no dietary-related difference.

**Table 2:** Lipid parameters in normal cholesterol chow diet and cholesterol-rich diet rats submitted to continuous moderate training (5 or 2 consecutive days/week) during 8 weeks

**Tissue/Group**	**Total Cholesterol (mg/dl)**	**Triglycerides (mg/dl)**	**HDL-cholesterol (mg/dl)**
**C**	62.17 ± 3.07	122.67 ± 15.09	18.67 ± 1.76
**TC5**	60.62 ± 3.11	127.00 ± 11.97	22.87 ± 1.27
**TC2**	66.12 ± 1.75	145.83 ± 4.70	23.57 ± 1.17
**H**	108.70 ± 5.22 °	177.83 ± 15.78 °	20.00 ± 1.26
**TH5**	78.00 ± 3.60 *	117.00 ± 12.40 *	24.85 ± 1.84
**TH2**	86.00 ± 4.43 *°	105.25 ± 4.99 * °	24.42 ± 1.28

* p ≤ 0.05 comparing trained × sedentary groups; +p ≤ 0.05 comparing 5 × 2 days/week exercised groups; ° p ≤ 0.05 comparing normal cholesterol chow diet × cholesterol-rich diet groups. Sedentary control (**C**) and cholesterol-rich diet group (**H**); 5 and 2× per week continuous training control (**TC5**) (**TC2**); 5 and 2× per week continuous training cholesterol-rich diet group (**TH5**) **(TH2).**

Table 3 shows that LIV and GAST relative weights in no situation suffered significant change because of diet or exercise.

**Table 3:** Relative weight of liver (LIV), gastrocnemius (GAST), retroperitoneal (RET) and epididymal (EPI) adipose tissue, and brown adipose tissue (BAT) in normal cholesterol chow diet and cholesterol-rich diet rats submitted to continuous moderate training (5 or 2 consecutive days/week) during 8 weeks

**Tissue/Group**	**LIV (%)**	**GAST (%)**	**RET (%)**	**EPI (%)**	**BAT (%)**
**C**	3.73 ± 0.07	0.48 ± 0.01	0.87 ± 0.06	1.23 ± 0.05	0.04 ± 0.01
**TC5**	3.67 ± 0.09	0.48 ± 0.01	0.51 ± 0.05 *	0.88 ± 0.03 *	0.12 ± 0.01 *
**TC2**	3.83 ± 0.11	0.49 ± 0.01	0.78 ± 0.06	1.18 ± 0.05	0.11 ± 0.01 *
**H**	4.69 ± 0.08	0.49 ± 0.01	1.09 ± 0.17°	0.90 ± 0.08 °	0.06 ± 0.01
**TH5**	3.92 ± 0.10	0.49 ± 0.01	0.64 ± 0.04 *	0.60 ± 0.03 *°	0.13 ± 0.01 *
**TH2**	3.92 ± 0.10	0.48 ± 0.01	0.79 ± 0.05*	0.68 ± 0.03 *°	0.08 ± 0.01 *^+*o*^

* p ≤ 0.05 comparing trained × sedentary groups; +p ≤ 0.05 comparing 5 × 2 days/week exercised groups; ° p ≤ 0.05 comparing normal cholesterol chow diet × cholesterol-rich diet groups. Sedentary control (**C**) and cholesterol-rich diet group (**H**); 5 and 2× per week continuous training control (**TC5**) (**TC2**); 5 and 2× per week continuous training cholesterol-rich diet group (**TH5**) **(TH2).**

Moderate continuous 5 days/week exercise significantly decreased EPI and RET relative weight in both diets when compared to respective sedentary groups on the same diet, while 2 days/week exercise (TC2) presented a decreased in RET and EPI when compared to sedentary control group (C), only in a cholesterol-rich diet.

Both 5 and 2 days/week exercise were effective in increasing BAT relative weight. However, the TH2 group showed no enhancement similar to that of the TH5 (^+^) and TC2(°) groups and, in response to the different diets, we found that the EPI relative weight in the hypercholesterolemic groups showed a decrease when compared to control groups (°) (Table 3).

Table 4 shows a significant increase in RET adipocyte area in H rats (°) when compared to the control group (C). The same change was not observed for EPI.

**Table 4:** Retroperitoneal (RET) and epididymal (EPI) adipocyte area in normal cholesterol chow diet and cholesterol-rich diet rats submitted to continuous moderate training (5 or 2 consecutive days/week) during 8 weeks

**Tissue**	**RET μm**^2^	**EPI μm**^2^
**C**	14619 ± 591.47	14809 ± 626.37
**TC5**	12115 ± 534.62 *	8413.9 ± 267.49 *
**TC2**	14768 ± 678.49 ^+^	16594 ± 766.18 *^+^
**H**	17058 ± 565.45 °	13059 ± 385.89
**TH5**	9243.6 ± 291.99 *°	8121.5 ± 218.36 *
**TH2**	11830 ± 414.32 *+°	11065 ± 334.52 *+°

* p ≤ 0.05 comparing trained × sedentary groups; +p ≤ 0.05 comparing 5 × 2 days/week exercised groups; ° p ≤ 0.05 comparing normal cholesterol chow diet × cholesterol-rich diet groups. Sedentary control (**C**) and cholesterol-rich diet group (**H**); 5 and 2× per week continuous training control (**TC5**) (**TC2**); 5 and 2× per week continuous training cholesterol-rich diet group (**TH5**) **(TH2).**

Continuous moderate 5 days/week exercise resulted in significantly decreased RET and EPI adipocyte area in both diets (*). We further observed that TH5 (°) group experienced a significant reduction in RET adipocyte area compared to TC5 group. Again, this was not the case for EPI.

No differences in values appeared for the RET adipocyte area when the TC2 group was compared to the C group, however, we found a significant difference when TC5 was compared to the TC2 group (^+^). On the other hand, the TH2 group presented a significant decrease when compared to H (*) as well as to the TC2 group (°) in RET and EPI adipocyte areas (Table 4).

## Discussion

Nutrition as well as exercise is known to play an important role in the etiology of hyperlipidemias, atherosclerosis, and metabolic disorders. In this study, we found that rats fed with cholesterol-enriched diet food intake values was higther than when compared to normal cholesterol chow diet groups (°) (Table 1). This data suggests that a diet enriched in cholesterol and cholic acid is more palatable then a normal cholesterol chow diet. Several studies have shown that a fat- and cholesterol-intake increase depends on social and cultural habits and lifestyle. It is also known that a strong correlation exists between this food type and lesser satiety coupled with greater palatability, which can result in much greater food intake and consequent dyslipidemias and/or obesity [4,10].

In addition, Table 1 demonstrates that a cholesterol-rich diet can significantly (H and TH5°) or relatively (TH2) increase body-weight gain, probably because of increased food (and, therefore, calory) intake, which because of the additional cholesterol with its properties, can cause dyslipidemias and obesity. However, 5 days/week continuous exercise both for normo or cholesterol-rich diet was effective to decrease weight gain. Two days/week exercised rats was effective only for the cholesterol-rich diet group, suggesting greater efficacy of the 2 consecutive times per week exercise protocol on hypercholesterolemia that for the normal cholesterol chow diet situation.

Comparing exercise frequencies we only verified modification in body-weight gain in normal cholesterol chow diet rats trained 5 days/week (^+^), since in this specific situation normal cholesterol chow diet rats trained 2 days/week experienced no body-weight decrease.

The food intake and body-weight gain results are very similar to those found by Deschaies et al. (1983) [10], that showed that exercise-trained rats ingested the same amount of calories as their sedentary counterparts, while the animals fed with a palatable diet consumed significantly more calories than the chow-fed groups.

Several animal and human studies have confirmed the hypercholesterolemic properties of a cholesterol diet, which include increasing TC, TG, and alterations in the lipoprotein pattern, the mechanisms of which remain under study [11-14]. In our study, we found that cholesterol diet was effective in significantly increasing TC and the TG fraction when comparing the H to the C group (°) but no significant differences were found for the HDL-c fraction.

Cholesterol homeostasis in the body is governed by an interplay of cholesterol absorption, synthesis, storage, and excretion. In human beings, plasma cholesterol levels may increase moderately through cholesterol addition to a baseline cholesterol-free diet. In rats, on the other hand, control mechanisms prevent a disturbance in homeostasis by reducing the body's cholesterol synthesis and by increased conversion of cholesterol to bile acids, leading to only a minor elevation of serum cholesterol after a feeding of large cholesterol quantities of [15]. However, cholic acid addition to the diet caused an increase in plasma total cholesterol levels, leading to other metabolic disorders and dyslipidaemias. In other words, the use of cholic acid to induce hypercholesterolemia by a cholesterol-diet in rats is essential, since cholic acid promotes cholesterol absorption, which was clearly shown in this study by the increase in the TC fraction (C × H°).

The significant TG plasma-concentration increases demonstrated in this study by the H group in comparison to the C group cannot be attributed only to the increase in cholesterol as primary source for the subsequently high TG synthesis, but probably also because of increased food intake. In studying the source of free fatty acids (FFA) and the pathways contributing to the accumulation of neutral fats, Liu et al. (1995) [11] showed a TG excess accumulated in livers of rats fed with a cholesterol-enriched diet (1% cholesterol in the diet for four weeks), the result of increased synthesis and decreased secretion of TG. Exercise has been widely recommended as an effective and non-pharmacological method for reducing CHD incidence, overall mortality, high blood pressure, as well as improving insulin resistance.

In this study, moderate continuous training, both 5 and 2 days/week, showed a statistical decrease in TC and TG levels in hypercholesterolemic, but not in normal cholesterol chow diet rats, suggesting greater efficacy of exercise in hypercholesterolemic situation. These data become more significant if we consider that a 1% decrease in plasma cholesterol can decrease by 2% the CHD mortality index [16]. Furthermore, in hypercholesterolemic rats both exercise frequencies (5 and 2 days/week) brought TC and TG concentrations closer to the values observed for the C group, but no significant differences were observed in the HDL fraction comparing different diets or exercise frequencies. These results are even better considering the existing consensus about increasing HDL-c fraction as a means of improving lipid profile [3,10,17].

Our results are very similar to others reported in the literature. Yan et al. (1997) [18], showed that sedentary rats fed with a high cholesterol diet had a higher level of serum TC compared with sedentary normal cholesterol chow diet rats, and rats fed on a high-cholesterol diet combined with continuous exercise had lower TC and higher HDL-c levels than sedentary hypercholesterolemic rats. Ensign et al. (2002) [19], demonstrated a significant drop in plasma TG concentrations, higher HDL-c levels, and lower plasma free fatty acids in a trained guinea pig group (7-week training program, 5 days/wk, 30–40 min per session) compared to a non-exercised group.

These studies, like ours, reveal a significant influence of moderate continuous exercise on lipids and lipoprotein fraction concentrations and composition, implying that exercise may enhance HDL-c fractions, and utilization and degradation of triglycerides and cholesterol, which increases the uptake of low-density lipoprotein and lowers plasma lipid levels, consequently underlining the role of exercise in the treatment and risk reduction of hypercholesterolemia and obesity [20-22].

In this study we found that even in normal cholesterol chow diet or cholesterol-rich diet situations, both exercise frequencies were effective in significantly decreasing EPI and RET relative weights, when compared to the sedentary groups. To cite only the TC2 group, we found a percentage drop of 10.34% and 4.06% for RET and EPI tissues respectively, when compared to the C group. Again, these results imply that continuous 2 days/week training is more effective in controlling fat accumulation in the hypercholesterolemic than in the normal cholesterol chow diet situation. In addition, other studies have found similar results for 5 days/week exercise protocols [23,24] but not for the 2 days/week training protocol, which may be important since a considerable adipose mass loss following a training period is a strong component in decreasing risk of CHD development in male adults [25].

It is known that hypercholesterolemic or high fat diets can promote enhancement of lipid synthesis and accumulation, which regular moderate exercise can minimize or control by causing an elevation in resting metabolic rate, lipid oxidation and degradation, as well as lipogenesis decrease [22,26].

Specifically as a result of the different diets used, we further noted that the relative weight of EPI in the H group remained the same in comparison with the C group. This result was unexpected since we had believed that cholesterol-rich diets could indirectly increase fat synthesis and accumulation, as was the case with RET tissue.

Both exercise frequencies statistically increased BAT relative weight. However, these changes were more pronounced in the groups that swam 5 days/week. Brown adipose tissue is known to be essential tissue in the mammalian thermogenic process. Several studies have questioned the hypothesis that exercise alone is enough to cause changes in this tissue, however, there is general agreement on the probability of changes occurring if exercise includes body-temperature maintenance, as does swimming [7,27]. Thus, the results found in this study agree with those of other studies such as that of Ueno e. al. (1997) [27], which showed that swimming training (1 h/day, 5 days/week, 6 weeks) significantly increased BAT mass and its protein content and indicating hypertrophy in both lean and obese mice,.

Liver and gastrocnemius relative weight changed significantly in none of the situations in response to exercise or diet. The reverse held for the GAST situation because a cholesterol-rich diet does not seem to enhance protein synthesis. Furthermore, using relative weight tissues muscle weight increase was compensated for increased total body weight. Similar results were found by Tulp & Jones (1987) [28] who, in studying the effects of increased energy expenditure on weight gain and adiposity in rats, demonstrated that exercise (less than 4 hr/day) produced no effect on the weight of either muscle tissue in lean or pre-obese rats.

Our results show that a cholesterol-rich diet increased significantly the adipocyte area in RET tissue of sedentary rats when compared to that of the respective control group, however, this was not the case for EPI. Although not an expected result, it agrees with EPI relative weight results showing no increase in sedentary hypercholesterolemic rats compared to the respective control group (Table 3). Furthermore, other studies using adult male rats have verified regional differences in the RET and EPI adipocyte area, with the EPI area proving smaller than the RET, which is a more central fat-pad then is EPI [29,30].

Lipid structure, composition, and configuration, in addition to excessive fat and cholesterol consumption, affect the lipid profile in plasma, as well as fat tissue deposition and gene expression of lipoproteins and their receptors [12]. It is known a cholesterol-rich diet can cause changes in lipid metabolism that result in dyslipidemias and probably a consequent increase in synthesis, storage, and accumulation of lipids in adipocytes. In the early of 70ths, in studying the regulation of cholesterol synthesis and storage in fat cells, Kovanen et al. (1975) [31] showed that rats fed on a high cholesterol diet presented a significant increase in serum and liver cholesterol. Thus, the cholesterol synthesis rate in fat cells was found to increase in close correlation with cell size. However, an increase in the TG fraction and its consequent incorporation into fat cells has also been shown to increase in a rough correlation with cell size. These results agree with a recent study by Portillo et al. (1999) [32] that demonstrated that a high cholesterol and fat amount in the diet induced adipose-tissue enlargement.

In this study, continuous exercise of 5 consecutive days/week resulted in a significant decrease in RET and EPI adipocyte area in both diets (*), while 2 consecutive days/week exercise showed a decrease only for the hypercholesterolemic group, again indicating favorable adaptations for this exercise frequency in a hypercholesterolemic situation. Fat oxidation is one of the lipid metabolism adaptations resulting from moderate continuous exercise. This adaptation occurs because the high-energy demand resulting from long-term exercise duration stimulates the release of the catabolic hormones that degrade lipids for use as an energy source [33].

Some studies observing the effects of 5 consecutive days/week swimming exercise in adult male rats report significant decrease in RET and EPI adipocyte area and diameters [23,30], but no mention was made of 2 consecutive days/week exercise frequency. Adipocyte area results generally showed that continuous training exercise (both 5 and 2 days/week) can promote a significant decrease in RET and EPI adipocyte area, mainly in hypercholesterolemic situations; however, the effects of 5 consecutive days/week training were more pronounced compared to those of 2 days/week training (^+^) (Table 4).

## Conclusion

The results found in this study show that moderate continuous exercise (both 5 and 2 consecutive days/week) can result in positive adaptations in adipocyte area and lipid parameters in normo- and hypercholesterolemic adult male rats. However, 5 d/wk traning promove more pronounced adaptations to contribute in preventing and controling hypercholesterolemia, dyslipidemias, and obesity.

## Methods

### Animals

Thirty-six adult male Wistar rats, weighing 220 ± 10 g and purchased from the Central Biotery of the Federal University of São Carlos, were housed in individual cages. The animals were kept at environmental temperature (23 ± 2°C) in a 12 h light/dark controlled room and randomly divided into six groups of six animals as follows: sedentary control (**C**) and cholesterol-rich diet group (**H**); 5× per week continuous training control (**TC5**) and cholesterol-rich diet group (**TH5**); 2× per week continuous training control (**TC2**) and cholesterol-rich diet group (**TH2**). This research was conducted in conformity with the Public Health Service (PHS) Policy on Humane Care and Use of Laboratory Animals. The Ethics Committee of the Federal University of São Carlos approved this protocol.

### Experimental procedure

After 8 weeks of treatment and training the animals were decapitated 24 hours after the last exercise session. Trunk blood collected in a heparinized tube was centrifuged for 15 min. at 2500 rev./min. Retroperitoneal and epididymal white adipose tissues, brown adipose tissue, liver, and gastrocnemius muscle were immediately removed, weighed, and frozen at -20° C for later biochemical and morphological analyses.

The relative weight of the tissues was calculated as follows:

Relative Weight (%) = (total weight of tissue/weight of rats) × 100

### Diet composition

#### Control diet

For standardization and definition of the average amount ingested by a group of adult male rats, two groups of 8 rats were previously kept sedentary for eight weeks in the same experimental conditions as those used for the present study. This procedure resulted in determining a control diet (NUVILAB^®^) containing 3.78 kcal/g., of which was offered to each group (C, TC5, TC2) 40 g/day, totaling 151.2 kcal/day.

#### Cholesterol-rich diet

The cholesterol-rich diet was prepared by adding to the standard diet (1%) 0.15% (p/p) of cholesterol Sigma^®^C 8503 diluted in ethyl ether P.A. stabilized with 10 ppm of butil hidroxi-tolueno-BHT, according to ROSSI et al. (2000) [34]. To make sure that the cholesterol would be absorbed, cholic acid Sigma^®^C 1254 (0.25%) diluted in absolute methanol Anhidro P.A. was also added. This diet was used from the beginning of the experimental period, for the purpose of inducing a hypercholesterolemic situation in the H, TH5, and TH2 groups.

#### Exercise protocol

Part of the animals were submitted to continuous moderate aerobic exercise (swimming) either 5 consecutive times a week (TC5 and TH5 groups) or 2 consecutive times a week (TC2 and TH2 groups) during an 8-week period. This was done in individual tanks (50 cm in height × 30 cm in diameter) in which the water temperature was maintained between 32–36° C and changed daily. A load of 5% in relation to rat body weight was tied on the tail to prevent floating and force activity throughout the period stipulated for the session. This load was recalculated weekly, following the procedure standardized by Dâmaso (1996) [7].

Adaptation time in accordance with the load and volume principles of training was as follows: on day 1 the rats swam for 30 minutes without load addition; day 2, thirty minutes with load addition; day 3, an hour and a half without load; day 4 and throughout the remaining days, an hour and a half with load (Dâmaso,1996) [7]. All rats adapted to the conditions imposed.

#### Weight control and food intake

Body weight and food intake of each animal were determined daily during the 8 weeks. Body weight gain, that means the percentage of increment of the initial body weight, was calculated by:

Body weight gain (%) = ((final weight – initial weight)/final weight) × 100

Food intake was defined as the weight difference between food offered and leftovers. The values obtained for each variable were taken during the whole experimental period and kept in individual records.

#### Plasma metabolites

Plasma was used to measure triglycerides (TG), total cholesterol (TC), and HDL-cholesterol. For these measurements we used commercial kits from Labtest Diagnostic S.A^®^, according to Guerrra et al. (2002) [35].

#### Adipocyte area determination

A fragment (100 mg) of retroperitoneal adipose tissue (RET) and epididymal adipose tissue EPI was separated and fixed in 0.2 M collidine buffer (pH 7.4) containing 2% of osmium tetroxide at 37°C. After 48 hours, they were washed with warmed saline, taken out, and spread on a plate as described by Hirsch & Gallian (1968) [36]. The adipocyte area was measured in different cells from the same tissue (EPI and RET) around 50 times using image analysis software (Image Pro Plus, KS-300, from Carl Zeiss) and expressed as μm^2^. The cells were randomly chosen and the analyser did not know to which group the cells belonged.

### Statistical analysis

Data were analyzed using two-way ANOVA to test: differences between exercised × sedentary animals fed on the same diet (*); differences in frequencies of exercise (5 × 2 times per week) in animals fed with the same diet (^+^); differences between the two diets (normal cholesterol chow diet × cholesterol-rich diet) at the same exercise frequency (°), following the Tukey-Kramer multiple comparison test. Statistic power was calculated and showed be above 0.8 for all cases.

## Abreviations

BAT = Brown Adipose Tissue

C = Sedentary Control Group

CHD = Coronary Heart Disease

EPI = Epididymal Adipose Tissue

FFA = Free Fatty Acid

GAST = Gastrocnemius muscle Tissue

H = Cholesterol-rich Diet Group

HDL-c = High Density Lipoprotein cholesterol

LDL-c = Low Density Lipoprotein cholesterol

LIV = Liver Tissue

PHS = Public Health Service

RET = Retroperitoneal Adipose Tissue

TC = Total Cholesterol

TC2 = 2× per week Continuous Training Control Group

TC5 = 5× per week Continuous Training Control Group

TG = Triglycerides

TH2 = 2× per week Continuous Training Cholesterol-rich Diet Group

TH5 = 5× per week Continuous Training Cholesterol-rich Diet Group

## Authors' contributions

RLFG: have been involved in design, data collected, drafting the manuscript and revising it critically for important intellectual content.

WLP, JPB, FPV, NCC: have been involved in data collected and drafting the manuscript.

LCV, IZC, EAR: have been involved in revising it critically for important intellectual content.

ARD: have been involved in design, drafting the manuscript and revising it critically for important intellectual content.
